# Olfactory Impairment and Recovery in Zebrafish (*Danio rerio*) Following Cadmium Exposure

**DOI:** 10.3390/biology14010077

**Published:** 2025-01-15

**Authors:** Chiara Maria Motta, Rosa Carotenuto, Chiara Fogliano, Luigi Rosati, Pabitra Denre, Raffaele Panzuto, Rossana Romano, Gianluca Miccoli, Palma Simoniello, Bice Avallone

**Affiliations:** 1 Department of Biology, University of Naples Federico II, 80125 Naples, Italy; mottacm@unina.it (C.M.M.); luigi.rosati@unina.it (L.R.); bice.avallone@unina.it (B.A.); 2 Department of Conservation of Marine Animals and Public Engagement, Zoological Station Anton Dohrn, 80122 Naples, Italy; raffaele.panzuto@szn.it; 3 Department of Sciences and Technology, University Parthenope, 80133 Naples, Italy; rossana.romano001@studenti.uniparthenope.it (R.R.); palma.simoniello@uniparthenope.it (P.S.)

**Keywords:** olfactory lamellae, behaviour, olfactory epithelium, collagen, carbohydrates, recovery

## Abstract

Anthropic activities have significantly elevated cadmium levels, making it a significant stressor in aquatic ecosystems. Present in high concentrations across water bodies, cadmium is known to bioaccumulate and biomagnify throughout the food chain. While the toxic effects of cadmium on the organs and tissues of aquatic species are well-documented, little is known about its impact on sensory systems crucial for survival. Consequently, this study investigated the impact of short-term exposure (96 h) to 25 µM cadmium chloride on the olfactory system of adult zebrafish. The research aimed to assess structural and functional changes in the zebrafish’s olfactory lamellae, providing a deeper understanding of how cadmium affects the sense of smell in this aquatic species. After exposure, cyto-anatomical alterations in the lamellae were analysed using light microscopy and immunocytochemistry. They revealed severe lamellar edema, epithelial thickening, and an increased number of apoptotic and crypt cells. Rodlet and goblet cells also increased by 3.5- and 2.5-fold, respectively, compared to control lamellae, and collagen density in the lamina propria increased 1.7-fold. Cadmium upregulated metallothioneins and increased the number of PCNA-positive cells. The olfactory function was assessed through a behavioural odour recognition test, followed by a recovery phase in which zebrafish exposed to cadmium were placed in clean water for six days. The exposed fish performed poorly, failing to reach food in five consecutive trials. However, lamellar damage was reduced after the recovery period, and their performance improved, becoming comparable to the control group. These results suggest that cadmium disrupts the sense of smell, and that recovery is possible after short-term exposure. This evidence sheds light on aspects of animal survival that are often overlooked when assessing environmental pollution.

## Simple Summary

Cadmium pollution, driven by human activities, is a significant stressor in aquatic ecosystems, bioaccumulating and biomagnifying in food chains. While its toxic effects on aquatic species’ organs are well-documented, its impact on sensory systems, such as the olfactory system, remains understudied. This research focused on short-term exposure (96 h) to 25 µM cadmium chloride in adult zebrafish, evaluating structural and functional changes in their olfactory lamellae.

## 1. Introduction

Cadmium pollution in water bodies, primarily from industrial activities (metallurgy, energy production), agriculture (agrochemicals), and sewerage runoff [1], poses a severe environmental threat. This toxic heavy metal accumulates in aquatic ecosystems, where it bioaccumulates and biomagnifies, affecting the health and survival of marine life [2]. Due to the high risk, cadmium concentration has been monitored in potentially polluted marine and freshwater bodies. Concentrations ranging from ng to µg are commonly registered [3,4]. Such values result from a progressive dilution of the metal: in the areas where the spill occurs, concentrations may rise to mg/L [5,6].

Cadmium, once internalised, accumulates in tissues [7], disrupting vital functions. In teleosts, the metal causes oxidative stress, dysregulates the immune system, and interferes with gene expression [8]. Consequent histopathological damage is observed in all tissues and organs, including the kidney [9], liver [10], skeletal muscle [11], and retina [12], to cite a few examples. Cadmium is also an endocrine disruptor since it interferes with reproduction, affecting the gonads, thyroid [13,14,15], and carbohydrate metabolism [16].

Less information is available on the response to short-term exposure at high concentrations, a situation registered in the case of an accidental spill. Laboratory tests on teleosts confirm that acute exposures, such as chronic ones, induce oxidative stress and immunotoxicity in the brain, ovary, and liver [17] and cause haematological, biochemical, and enzymological alterations [18].

In fish, the paired olfactory organs consist of olfactory lamellae organised in a rosette. It is housed in a nasal cavity connected to the exterior via two nostrils [19]. Due to their position and the continuous water flow, the lamellae are an easy target for toxicants [20,21], including cadmium [22]. The metal alters gene expression [23], causing histopathological alterations [22] that affect the complex organisation of the sensory and non-sensory epithelium [19]. The consequence is a serious threat to the animal’s survival. The olfactory deficits may impair the search for food and social interactions, including reproduction and the escape from predators [20,24,25].

Therefore, the present study’s objective was to provide the basis for a more accurate and comprehensive evaluation of the impact exerted by acute cadmium exposure. Characterised by a prominent methodological perspective, this study analysed the effects of exposure to 25 µM CdCl_2_ for 96 h on the olfactory response of *Danio rerio*, a key model for studying the effects of toxicants in teleosts [26]. The Cd concentration was chosen according to Motta et al. [27].

The effects on behaviour were investigated in young males since it is known that the olfactory response markedly differs among the sexes [28]. An odour detection test was conducted in a labyrinth tank [29]; it determined the time employed to reach the food, the time the fish spent exploring the different areas of the labyrinth, and the short-term memory.

Effects on behaviour were correlated to the cytotoxic effects of the metal on the olfactory lamellae. Conventional light microscopy (haemalum–eosin staining) and Picrosirius Red staining highlighted the possible interference on general anatomy, including collagen deposition in the connective lamina propria [30]. PAS, Alcian Blue, and phalloidin specifically stained goblet (mucus) and rodlet cells, allowing counting and a rough estimation of tissue inflammation [31,32] and stress [16,33]. The activation of tissue defence and recovery mechanisms was evaluated by detecting the expression of metallothioneins (MTs; [27]) and proliferating cell nuclear antigen (PCNA; [34]). Finally, since cadmium interferes with carbohydrate metabolism [16] and carbohydrates are involved in olfactory response [35], the lamellae’s carbohydrate composition was investigated by staining with the lectin WGA, specific for N-Acetyl glucosamine [27,36].

All behavioural and cytological tests were repeated after six days of permanence in clean water to test recovery, an aspect neglected in the literature but deserving attention. In teleosts, studies mostly deal with cadmium elimination from intoxicated tissues [37,38], while less evidence is available regarding recovery from toxic effects. It is demonstrated that some, like anaemia and hypermagnesemia, disappear more rapidly than others, for example, hypocalcaemia. On the contrary, hyperglycemia, present during the exposure, persists throughout the recovery period [39], while in testes, reversal after cadmium withdrawal is very limited [40]. Recovery from contamination, therefore, deserves more investigation.

## 2. Materials and Methods

### 2.1. Zebrafish Maintenance and Exposure to Cadmium Chloride

Four-month-old male fish (n = 96; average wet weight 1.20  ±  0.2 g), maintained at 25 ± 1 °C, pH 7.0 ± 0.1, and a natural photoperiod, were randomly allotted into eight 30 L tanks (n = 12 animals/tank). They were fed twice daily [41] with commercial pellets produced for small freshwater teleosts. At treatment, four tanks were left untreated (experimental control, n = 48 fish), and four were exposed to 25 µM of CdCl_2_ [27] (n = 48 fish) under static conditions (no CdCl_2_ was added during the experimental period). No mortality or signs of suffering (rapid opercular movements, erratic swimming, or abrupt changes in direction) were recorded during the treatment. The concentration chosen (4.6 mg/L) was below the estimated 96 h exposure LC_50_ value that ranges from 5.0 [10] to 9.8 [42] mg/L.

After 96 h of treatment, all the animals were used in the behavioural tests (n = 48 control fish + 48 Cd fish). In the end, three animals per tank were sacrificed for histological analyses (Cd 96 h, n = 12 control fish + 12 Cd fish), while the remaining nine animals/tank were transferred into uncontaminated water and allowed to recover. During recovery, the water was changed daily to discard the excreted cadmium. After six days, all the animals in the four control and four Cd exposed tanks were used in the behavioural tests (n = 36 control fish + 36 Cd fish), and then three animals per tank were sacrificed for histological analyses (Cd 96 h recovery, n = 12 control fish + 12 Cd fish). The experimental plan is reported in Figure S1.

The experiments were carried out in compliance with the ethical provisions established by the EU Directive 2010/63/EU for animal experiments and according to the “Guideline for Animal Experimentation” of the Italian Department of Health. They were carried out in the Department of Biology Animal Facility and organised to minimise stress and the number of animals used. The University of Naples Federico II Animal Care Review Board approved all the experiments.

### 2.2. Behavioural Tests

**Figure 1:**
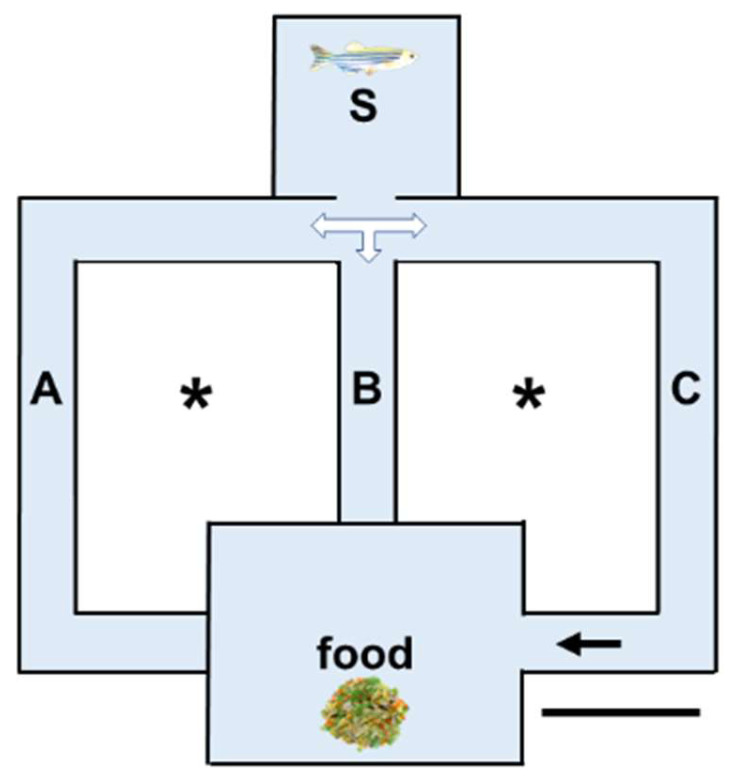
Schematic representation of the labyrinth tank used for testing the olfactory response. Starting chamber (S), closed corridors A and B, open corridor C to access the food chamber. Tanks filled with heated water (*). Bar: 20 cm.

After fasting for 36 h [43], the animals were individually transferred to a glass labyrinth (approximately 70 × 70 × 12 cm; Figure 1) containing uncontaminated water maintained at 25 ± 2 °C thanks to two central reservoir tanks containing heated water. The labyrinth was placed in a quiet room under a diffused light, and the same orientation was maintained (the tarting chamber was positioned to the north). At the beginning of the test, the fish was placed in the starting chamber (S); at the same time, food was added to a food chamber that could be reached only by swimming along a long corridor (C). Another two corridors (A and B) were closed at the end. For food, we used commercial pellets for fish containing animal proteins to ensure the presence of attracting amino acids. In the experiments, we consistently used the same preparation to activate the same subset of olfactory receptors [44].

A camera was placed above the labyrinth, and all the trials were recorded and examined later with Tracker Online to retrieve data. For each trial, the following was determined: (1) the time initially spent exploring the starting chamber; (2) the time employed by the fish to reach the food; and (3) the time spent by the fish in the different corridors and, if the case, back in the S chamber during the trial. Times were calculated from when the fish snout entered a chamber to when the tail left the chamber. The rationale was that by progressing in the C corridor, changes in odour would be detected if the lamellar function was not reduced by Cd exposure. Each animal was afforded five trials, each lasting a maximum of 5 min, separated by a period of 15 min of rest [45] during which it was maintained in a separate tank. During the rest period, the water in the labyrinth was renewed.

### 2.3. Olfactory Lamellae Sampling, Processing, and Staining

The animals were sacrificed by immersion in chilled water containing an overdose of ethyl 3-aminobenzoate methanesulfonate (MS-222, 300 mg/L; Sigma Aldrich, Milano, Italy). Heads were dissected and immediately fixed in Bouin’s solution (6 h), dehydrated in ethanol, and embedded in paraffin wax. Sections (6 μm) were mounted on glass slides and stained with haemalum–eosin to show general morphology or FITC-WGA lectin, highlighting glucNAc in goblet cells [27]. To reveal collagen alterations in lamellar lamina propria, sections were stained with Picrosirius Red (0.1 g Direct Red 80 from Merck dissolved in saturated picric acid) and observed under bright light. The density of the stained lamina propria was quantified by determining the optical density (see Section 2.5). Goblet cells were marked by staining with Alcian Blue [46], while rodlet cells were detected after staining with PAS [47]. Their presence and increased number were confirmed by staining apical actin with fluorescent phalloidin [33].

### 2.4. Immunolocalization of PCNA and MT

Sections were heated in citrate buffer pH 6.0 to unmask the antigens and then incubated in 2.5% H_2_O_2_ to block the endogenous peroxidase and normal goat serum to reduce non-specific bindings. The sections were incubated overnight at 4 °C with the primary antibody (rabbit anti-human PCNA, 1:300, Elabscience, Houston, TX, USA; rabbit anti-mouse MT, dilution 1:200; Santa Cruz Biotechnologies, Inc.; Santa Cruz, CA, USA). The next day, the sections were incubated with HRP-conjugated goat–anti-rabbit secondary antibody (1:200 in normal goat serum) for 1 h at room temperature and then incubated with avidin-biotin-peroxidase complex (ABC immune peroxidase kit, Pierce, VWR International, Milan, Italy) for 1 h at room temperature. Binding was revealed with diaminobenzidine (DAB). Negative controls were prepared by omitting the primary antibody or pre-digesting sections with protease [26]. Positive controls were prepared by immunostaining the retina (MT; [12]) and the testis (PCNA; [34]).

### 2.5. Morphometric Measurements, Cell Counts, and Determination of Staining Intensity

Random digital photos (20× magnification) were taken from the lamellae of control, treated, and recovered animals. They were then examined using ImageJ software (free version 1.8.0; updated 22 May 2023; https://imagej.net/ij/download.html (accessed on 10 January 2025)). In particular, the height of the olfactory epithelium (sensory and non-sensory; [34]; n = 50 measures/treatment) was determined from the basal lamina to the top of the epithelial cells, excluding microvilli and cilia. Numerical data obtained were pooled and reported as a percent of the control values.

The goblets and rodlets cells were counted in Alcian Blue- or PAS-stained sections (n = 50 counts/treatment). The same procedure was adopted to count PCNA-positive cells. Variations in collagen were quantified in Picrosirius Red-stained lamellae by using ImageJ software (see above). Images (20× magnification) were converted into high-resolution (400 dpi), 8-bit, 256 grayscale TIFF images. Density was determined in n = 50 square areas/treatment, selected in correspondence with the lamina propria, bordered by one tract of basal lamina. Numerical data obtained were pooled and reported as a percent of the control values.

### 2.6. FITC-WGA Staining

Sections were stained in the dark with 1 µL of FITC-WGA lectin (2 mg/mL), diluted in 30 µL of PBS (0.2 M, pH 7.2–7.4). After 10 min in a humid chamber, slides were rinsed in PBS, and sections were observed under UV light at 495–500 nm, as indicated by the manufacturer (Vector Laboratories Inc., Burlingame, CA, USA). Autofluorescence was first assessed by examining unstained sections. Controls were prepared by incubating the lectin with the competing sugar; positive controls were represented by tissues of known positivity to the lectins present in the same section [27,36]. Labelling was defined as positive or negative by the same observer.

### 2.7. Statistical Analysis of Data

The significance of treatments was assessed using a Student’s *t*-test, and one-way and two-way ANOVAs followed by a Tukey’s pairwise comparison test. Statistical analyses were conducted using GraphPad Prism 9.0 Software (San Diego, CA, USA). The minimum significance level accepted was *p* < 0.05 (*). Cohen’s d was used to determine the size effects of treatments; in particular, d = (x1 − x2)/s, where x1 and x2 are the sample means of group one and group two, and “s” is the standard deviation of the population from which the two groups were taken [48].

## 3. Results

### 3.1. Behavioural Tests: Control and Cadmium-Treated Animals

**Figure 2:**
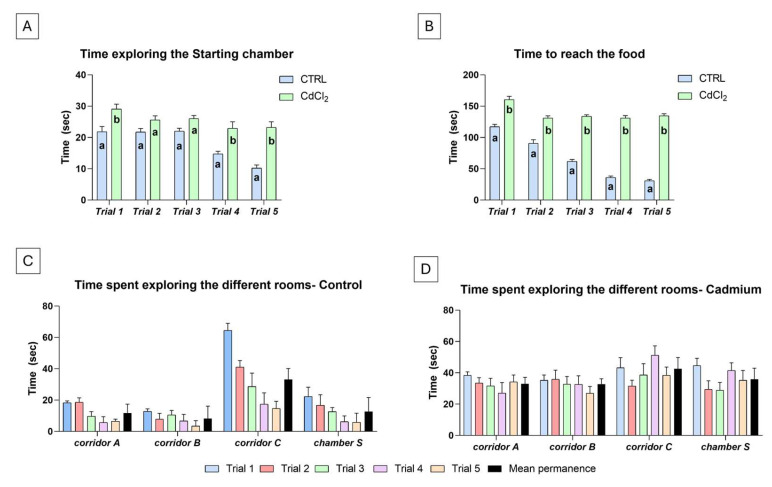
Response in a five-trial odour test of cadmium-treated *Danio rerio*. A significant decrease in time spent exploring the starting chamber S (**A**) and reaching food (**B**) is observed in the controls but not in treated animals. Different letters indicate significant differences from the corresponding control. (**C**,**D**) Time spent exploring the different areas of the labyrinth. Two-way ANOVA followed by a Tukey’s pairwise comparison test.

In the controls, the average time spent exploring the starting room S (Figure 2A) was 18.1 ± 4.8 s; a significant reduction in time was observed from the first (21.8 ± 4.3 s) and the fifth (10.2 ± 2.1 s) trial. In Cd-treated animals, the average time was significantly higher (25.4 ± 2.2 s; *p* < 0.0001) compared to the control, and though performance improved in trials 2 to 5, the maximum (29.1 ± 4.5 s) and the minimum (22.9 ± 2.0 s) values were recorded in the first and fourth trial, respectively.

The average time employed by the control fish to explore the labyrinth and reach the food was 67.4 ± 16.6 s (Figure 2B). Times reduced significantly in subsequent trials (from 117.3 ± 12.7 s in the first trial to 30.9 ± 3.5 s in the fifth trial). In Cd-treated animals (Figure 2B), the situation was completely different. The average time to reach the food was 138.2 ± 18.4 s, with a maximum of 160.4 ± 15.4 s in the first trial and a minimum of 131.2 ± 7.9 s in the second trial. Times did not change significantly in the following trials.

On average, in the five trials, control animals (Figure 2C) randomly entered one of the three corridors after leaving the starting room S. If they entered corridor A or B, they rapidly turned and came out (average permanence was 11.8 ± 5.7 and 8.2 ± 7.9 s, respectively). If they entered corridor C, they explored cautiously and suddenly swam to the food. The average permanence in this corridor was 33.2 ± 6.8 s. Occasionally, the animals returned to the starting room S (average permanence, 12.7 ± 4.9 s). In subsequent trials, the time spent in the corridors and S chamber reduced significantly.

Cadmium-treated animals (Figure 2D) showed a completely different behaviour. As with the controls, once leaving the starting chamber S, the fish indifferently entered one of the three corridors, but on average, once inside, they swam without an apparent destination, often coming back even if already very close to the food. The average permanence time in a corridor ranged from a minimum of 32.7 ± 4.4 s in corridor B to a maximum of 42.6 ± 7.3 s in corridor C. In addition, fish tended to re-explore the starting chamber, remaining inside for an average of 35.9 ± 6.3 s. The time spent in the corridors and S chamber was not significantly reduced with trials.

### 3.2. Behavioural Tests: Control and Cadmium-Treated Animals After Six Days of Recovery

**Figure 3:**
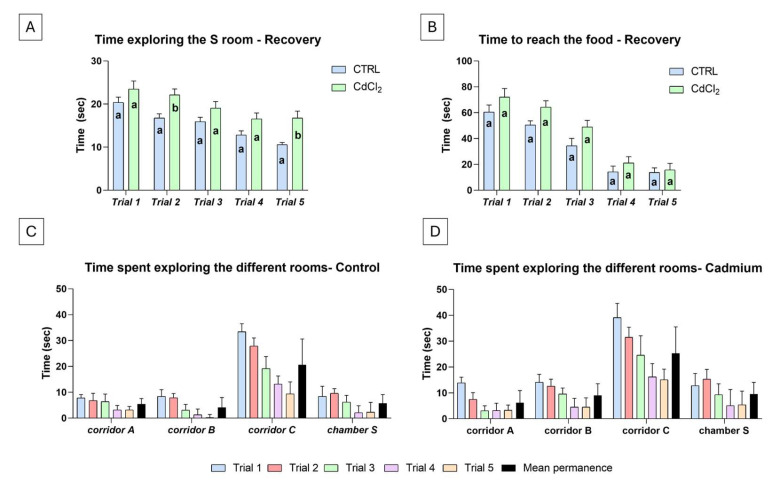
Response in a five-trial odour test of cadmium-treated *Danio rerio* after a 6-day recovery in uncontaminated water. A significant decrease in time spent exploring the starting chamber S (**A**) and reaching the food (**B**) is observed in both the control and Cd-treated animals. Different letters indicate significant differences from the corresponding control. (**C**,**D**) Time spent exploring the different areas of the labyrinth. Two-way ANOVA followed by a Tukey’s pairwise comparison test.

After six days in clean water, in the controls, the average time spent exploring the starting room S (Figure 3A) was 15.3 ± 4.3 s; a significant reduction was observed from the first (20.4 ± 3.6 s) to the fifth (10.6 ± 2.3 s) trial. In Cd-treated animals, the average time was significantly higher (19.6 ± 5.8 s) compared to the controls; performances, however, significantly improved with trials since the first and fifth trials recorded the maximum (23.5 ± 3.2 s) and minimum (16.6 ± 3.7 s) values, respectively.

The average time employed by the control fish to explore the labyrinth and reach the food was 34.7 ± 4.5 s (Figure 3B), with a significant reduction observed from the 60.5 ± 5.4 s of the first trial to the 13.7 ± 2.1 s of the fifth trial. The behaviour in Cd-treated animals (Figure 3B) was similar: the average time to reach the food was statistically higher than that of the controls (44.4 ± 5.3 s). However, as in the controls, performances significantly improved with trials. Times were reduced from an average of 72.1 ± 6.4 s in the first trial to a minimum of 15.7 ± 3.0 s in the fifth trial, a time not different from that of the relative control.

In the five successive trials, control (Figure 3C) and recovered (Figure 3D) animals randomly entered one of the three corridors after leaving the starting room S. In both cases, if they entered corridor A or B, they rapidly came out (average permanence less than 10 s). In contrast, if they entered corridor C, they explored carefully and rapidly progressed towards the food. The average permanencies in this corridor were 20.6 ± 3.8 and 25.3 ± 9.2 s, respectively. Occasionally, the animals returned to the starting room S, but the average permanence reduced to 5.7± 3.4 and 9.6 ± 4.5 s, respectively. In subsequent trials, the time spent in the corridors and S chamber was always significantly reduced.

### 3.3. Effects of Cadmium and Recovery on the Anatomy of the Olfactory Lamellae

**Figure 4:**
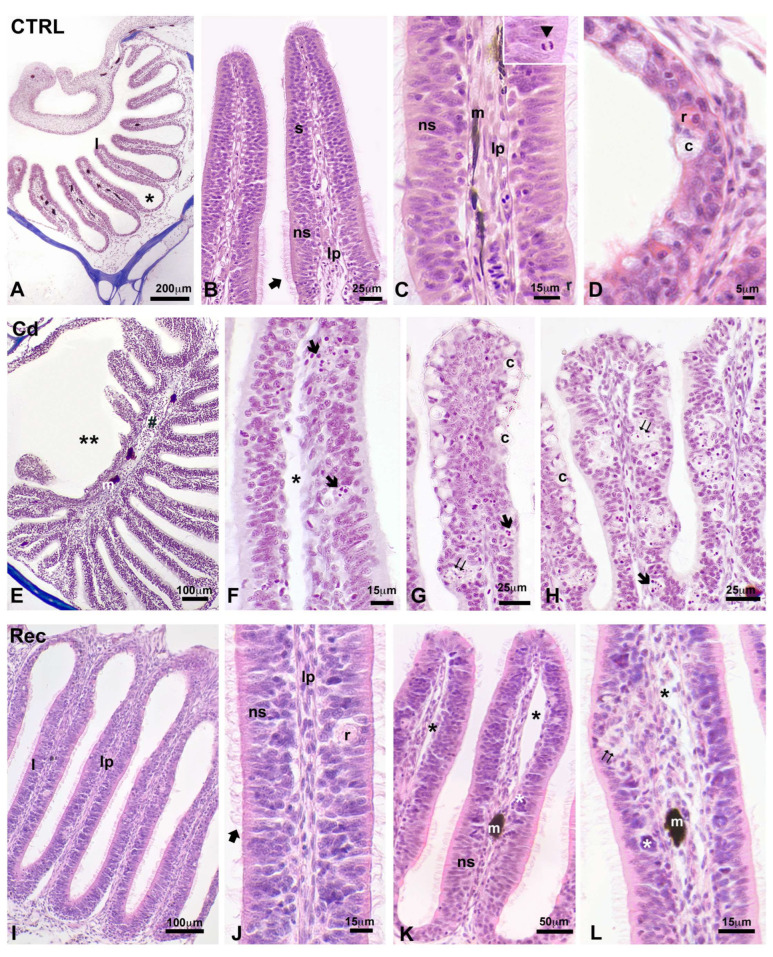
Microscopic anatomy of the olfactory lamellae in the control (**A**–**D**), cadmium-treated (**E**–**H**), and recovered (**I**–**L**) *Danio rerio*. (**A**) Olfactory rosette in the nasal chamber. Lamellae (l) and lateral channel-like system (*). (**B**) Detail of polymorphic sensory (s) and non-sensory (ns) epithelium, and lamina propria (lp). Note the ciliated non-sensory epithelium (big arrow). (**C**) Further detail of ciliated non-sensory (ns) epithelium, lamina propria (lp), and melanocytes (m). The apical portion of the epithelium contains rodlet cells (r) and mitotic figures (arrowhead in the inset). (**D**) Detail of a crypt (c) and rodlet (r) cell in the channel epithelium. (**E**) Altered lamellae (**) and increased melanocytes (m) in the median raphe (#). (**F**) Detail of a moderately altered lamella; notice the apoptotic bodies (arrow) in the epithelium and the oedematous lamina propria (*). (**G**,**H**) Groups of altered cells (double arrow) with apoptotic bodies (arrow) and increased number of crypt cells (c). (**I**,**J**) Intact lamellae (l) with non-sensory epithelium (ns) showing well-organised cilia (big arrow), lamina propria (lp), and rodlet cell (r). (**K**,**L**) Oedematous lamina propria (*) and altered epithelium (white *). Presence of melanocytes (m). Haemalum–eosin staining. Bars: 200 µm (**A**); 100 µm (**E**,**I**); 50 µm (**K**); 25 µm (**B**,**G**,**H**); 15 µm (**C**,**F**,**J**,**L**); 5 µm (**D**).

The olfactory organ consisted of a nasal cavity containing an olfactory rosette, a paired series of lamellae inserted in a midline raphe. The youngest was shorter and located anteriorly (Figure 4A). The lamellae were covered by epithelium, about 25 µm thick (Figure 4B), in which the olfactory receptor cells, supporting cells, and small basal cells were closely packed and not organised in layers (Figure 4C). This pseudostratified epithelium lay on a basal lamina and a connective lamina propria containing capillaries and nerves and occasional pigment cells (Figure 4C). Goblet cells were rare, as were crypt sensory neurons and rodlets cells (Figure 4D).

**Figure 5:**
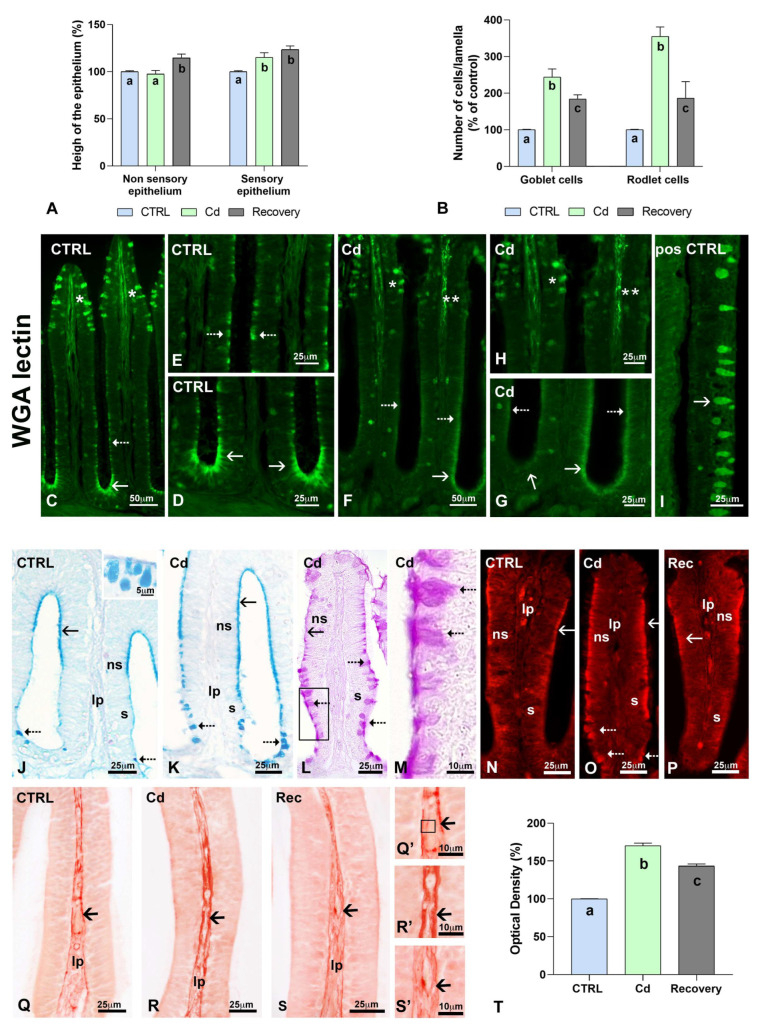
Characterization of the olfactory lamellae in control, cadmium treated and recovered *Danio rerio*. (**A**) Increased height of non-sensory and sensory epithelia. (**B**) Increased number of goblet and rodlet cells. (**C**) Labelled goblet cells (*) and apical cytoplasm in non-sensory (dotted arrow) and ring channel epithelia (arrow). (**D**,**E**) Details of (**C**). (**F**) Labelled (*) and poorly labelled (**) goblet cells. Labelled and poorly labelled apical cytoplasm of lamellar (dotted arrow) and ring channel (arrows) cells. (**G**,**H**) Details of (**F**). (**I**) Positive control; labelled skin goblet cells (arrow). (**J**,**K**) Alcian Blue-stained goblet cells (dotted arrows) and microvilli (arrows). (**L**) PAS-stained rodlet cells (dotted arrows) and apical cytoplasm of non-sensory cells (arrow). (**M**) Detail of (**L**) (frame). (**N**–**P**) Phalloidin stain; positive actin-rich apical cytoplasm in sensory (dotted arrows) and non-sensory (arrow) epithelial cells. (**Q**–**S**) Picrosirius Red-stained collagen in the basal membrane of the epithelium (arrows) and lamina propria (lp). (**Q’**–**S’**) Details of (**Q**–**S**). (**T**) Optical density (grey values) measured in the areas of the lamina propria indicated in the frame in (**Q’**). n = 50 measures/treatment. One-Way ANOVA followed by a Tukey’s pairwise comparison test. Different letters indicate statistically significant differences among groups. Bars: 50 µm (**C**,**F**) 25 (**D**,**E**,**G**–**S**) 10 µm (**M**,**Q**’–**S**’). Non-sensory (ns, ciliated)/sensory (s) epithelia; lamina propria (lp).

After cadmium exposure, the olfactory organ was occasionally altered (Figure 4E). In general, the lamina propria was oedematous (Figure 4F), and the goblet, rodlet, and crypt cells were significantly increased in number (Figure 4G and Figure 5B). The epithelium often detached from the underlying connectives (Figure 4F) and showed disorganised areas, characterised by the presence of groups of cells with pale cytoplasm and/or pyknotic nuclei and/or apoptotic bodies (Figure 4H). After six days of recovery in clean water, the lamellae appeared almost normal in morphology (Figure 4I): the epithelia were better organised (Figure 4J), and the lamina was only occasionally oedematous (Figure 4L).

Morphometric analyses did not evidence differences among the control animals (group examined at 96 h and group examined after the six days of recovery). In contrast, analyses provided significant information about the extent of the alterations observed in Cd-treated lamellae. Measures demonstrated that cadmium increased the height of the sensory but not the non-sensory epithelia (98.7 and 123.6% of the control; Figure 5A). After a 6-day recovery, the height further increased in both sensory and non-sensory epithelia, with values reaching 126.4 and 139.5% of the control, respectively (Figure 5A). The Cohen’s d values for the effect size were 1.5 for non-sensory epithelium and 1.7 and 2.7 for sensory epithelium, treated and recovered, respectively.

Goblet cells were counted in Alcian Blue-stained lamellae (Figure 5J,K). Numbers increased by 144.2% after Cd exposure and reduced to 84.4% after recovery (Figure 5B). In control lamellae, goblets were intensely stained by WGA lectin (Figure 5C). The lectin also stained the apical cytoplasm of scattered cells in non-sensory epithelia (Figure 5E) and the apical cytoplasm of ring channel epithelial cells (Figure 5C,D). After Cd exposure, several goblets showed reduced labelling (Figure 5F–H). At the same time, labelling on the apical cytoplasm of sparse epithelial cells tended to disappear (Figure 5F), as did labelling on ring channel epithelial cells (Figure 5F,G). In recovered animals, labelling distribution and intensity did not differ significantly from the control animals.

Rodlet cells also increased in number, as indicated by phalloidin staining (Figure 5N–P) and counting in PAS-stained lamellae (Figure 5L,M). Their number increased 235.1% after cadmium exposure and reduced to 86.5% after recovery (Figure 5B). The most significant Cohen’s d values for the effect size were registered after Cd exposure (for rodlets, 4.9; for goblets, 2.2), while after recovery, the effect sizes were reduced to 1.8 and 1.5, respectively.

Quantitative analyses of the lamina propria stained with Picrosirius Red confirmed the alterations observed after staining with haemalum–eosin. The absorbance demonstrated a 170.1% increase in collagen (Figure 5T) after cadmium exposure (Figure 5R,R’). After recovery in clean water, collagen appeared more dispersed and homogenously distributed (Figure 5S,S’), though the absorbance values were still 143.6% higher than those measured in controls (Figure 5T). The Cohen’s d values for the effect size for Cd-treated and recovered samples were 4.9 and 2.5, respectively.

### 3.4. Immunodetection of MT and PCNA

**Figure 6:**
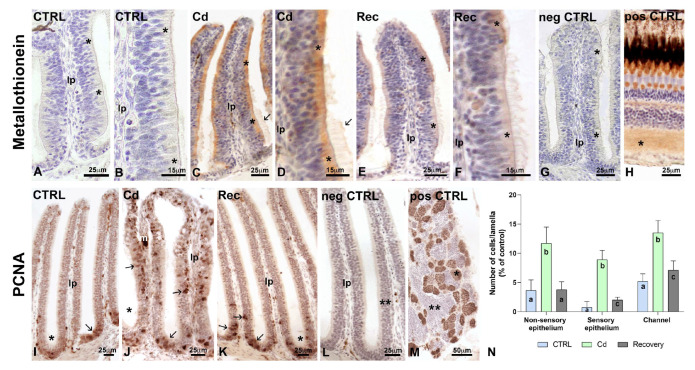
Metallothionein and PCNA expression in the olfactory lamellae in control, cadmium-treated, and recovered *Danio rerio*. (**A**,**B**) No MT is evident in the cell cytoplasm (*). (**C**,**D**) Increased MT expression (*). Notice staining in cilia (small arrow). (**E**,**F**) Reduced MT expression (*). (**G**) Negative control; unstained epithelium (*). (**H**) Positive control; stain on inner plexiform-layer cytoplasm (*). (**I**–**K**) Localization of PCNA-positive cell nuclei (small arrows). (**L**) Negative control; unstained epithelium (**). (**M**) Positive control; stain on the spermatocytes. Stained cysts (*), unstained cysts (**), lamina propria (lp). Bars: 50 µm (**M**); 25 µm (**A**,**C**,**E**,**G**–**L**) 15 µm (**B**,**D**,**F**). (**N**) Variation in the number of PCNA-positive nuclei in non-sensory, sensory, and channel epithelia. Staining with peroxidase-conjugated antibodies; nuclei counterstained with haemalum. *, *p* < 0.01; n = 50 lamellae/treatment. One-Way ANOVA followed by a Tukey’s pairwise comparison test. Different letters indicate statistically significant differences among groups.

MT, absent in control epithelia (Figure 6A,B), markedly increased in the cytoplasm of cadmium-exposed lamellae (Figure 6C,D). After recovery, staining reduced significantly (Figure 6E,F).

In control epithelia (Figure 6I), PCNA-positive cell nuclei numbered 3.6 ± 1.8 in non-sensory and 0.75 ± 1.3 in sensory epithelia (Figure 6N). Positive nuclei were more numerous in the ring channel epithelium, reaching an average of 5.2 ± 1.3 per lamella (Figure 6N). After cadmium exposure (Figure 6J), stained nuclei increased significantly, ranging from a minimum of 8.9 ± 1.6 in the sensory epithelium to a maximum of 13.5 ± 2.1 in the ring channel epithelium (Figure 6N). Recovery in clean water restored the control condition in non-sensory epithelia, while in sensory and ring channel epithelia, values reduced to 2.0 ± 0.5 and 7.1 ± 1.6 (Figure 6N). The Cohen’s d values for the effect size of cadmium samples were always ˃4.5. After recovery, they reduced significantly, ranging from a minimum of 0.5 in non-sensory epithelium to a maximum of 2.1 in sensory epithelium.

## 4. Discussion

Teleost fish use chemosensing to gain information about the surrounding environment [49]. The olfactory system can perceive and discriminate various odours [50]. During evolution, this ability led to the development of distinctive behavioural responses, such as the escape from predators [51] and the search for a partner [52] or food [53]. These innate olfactory-driven behaviours, also present in animals that have only experienced caged life, such as trout [54], can be consolidated by conditioning [55,56]. The labyrinth tank was designed to activate such mechanisms and test cadmium’s interference.

In our experiments, the initial cautious exploration of the labyrinth demonstrated that control fish in captivity retained the antipredator behavioural response, particularly a careful approach during the inspection of the novel environment [57]. When food odour was first perceived, the appetitive swimming response [58] drove the animal to the food reward, consolidating conditioning and improving performances with trials [55,56,59]. As expected [60], Cd interfered with such behaviour, and according to the literature, this would depend on interference with olfactory lamellae but not with the mouth chemosensing as demonstrated in *Danio rerio* [56].

The slowdown of performances after Cd exposure should not depend on direct interference in swimming. In *Cyprinus carpio* and Nile tilapia, it has been demonstrated that skeletal muscle is a poor Cd-accumulating tissue [61,62]. In *Danio rerio*, at a low concentration, it takes 7 days of exposure to alter gene expression (cyt, bax, gadd, and rad51; [63]); therefore, it is improbable that 96 h were sufficient to determine damages so severe to affect swimming [11]. Indeed, during the experimental period, fish showed no typical signs of behavioural alterations such as bottom-dwelling or freezing [64].

Similarly, the involvement of the olfactory bulbs in the delayed behavioural response is unlikely. Lower levels of cadmium have been registered in the bulbs compared to the olfactory mucosa [65]. In addition, it has been shown that cadmium diffuses in the bulbs already complexed with MT [66]. In contrast, interference in behaviour via the olfactory system may be extremely fast. Lamellae offer a wide surface for direct contact with contaminated water, and the microvilli and long cilia of olfactory neurons further increase the exposed surface. Not surprisingly, Cd accumulates in the olfactory epithelium [67].

In both adult and larval teleosts, besides altering behaviour [68], cadmium impairs physiological [69] functions and causes cell stress [70]. In our experiments, the first evidence of tissue stress was the increased number of goblet cells. This occurs in all mucous membranes, in gut villi [27], gill lamellae [71], and skin [72]. As a component of the innate immune system, mucus binds toxic cadmium and avoids ion loss from the damaged epithelium, exerting a protective role [72].

In *Danio rerio*, no increase in mucus was observed in the nasal chamber. The continuous water flow probably washed it away, on one side leaving the epithelial surface accessible for the olfactory functions but, on the other, increasing contact with the metal and damage.

The increase in rodlet cells is another evidence of the activation of defensive mechanisms. Though their function has not been fully characterised in teleosts, rodlets have been implicated in ion transportation, inflammation, and immune response [73]. Their increase in the tissue is a marker of stress, no matter the origin [74]. From this point of view, the data on rodlets correlates well with the observed increase in goblet cells and the subsequent reduction when exposure to cadmium ended. The two cell types, therefore, collaborate in epithelial defence as expected, considering that rodlet cells have also been implicated in mucus glycoprotein elaboration [75].

Cd penetration in the tissues also activates regulatory mechanisms that change physiological parameters, for example, cortisol levels [76], initiating downstream molecular and cellular responses [77]. Among these, cadmium activates the expression of MT, small proteins specialised in chelating metals, including cadmium [78]. In *Danio rerio*, MT expression increases as early as after 24 h of exposure, and increased MT proteins have been detected in several tissues [12,27], including in larval *Danio rerio* [79]. However, as demonstrated in pike, glutathione would be the first ligand for cadmium [66].

MT markedly increased in our experiments, localising in the apical cytoplasm of epithelial cells and cilia, the most exposed areas, due to the high surface/volume ratio. Antioxidant genes (hmox1 and prdx1) and heat shock proteins [23] were also activated, even at low Cd concentrations, to defend tissues from Cd-induced lipid and DNA peroxidation [80]. Sensory neurons are susceptible to metal-induced oxidative stress since their membranes are rich in ion transporters, with which divalent cation Cd competes [81]. The obvious consequence is a cascade of degenerative events [17], ending in apoptosis and a loss of function, and in anosmia in our specific case.

Light microscopy did not allow for determining the extent of damage to neurons or for characterising which were the most affected. Ciliated neurons should be the most vulnerable due to their large surface being exposed to metal; microvillar neurons also seem to be affected, as changes in Alcian Blue and lectin staining suggested. Ongoing investigations at the electron microscope will clarify the damage that occurred while immunocytochemistry, thanks to specific antibodies, will allow for discrimination on different sensory and non-sensory cells [82]. In teleosteans, the olfactory epithelium contains four different types of sensory neurons [83], and these subpopulations may respond differently to toxicants [34] and different odorant classes depending on the existence of differential projections to the olfactory bulb [84]. We have no information in this regard since, for now, only a commercial food pellet containing several different components was used in the experiments.

Cell damage, however, did occur in the olfactory epithelium, as indicated by the presence of many disorganised areas, apoptotic bodies, pyknotic nuclei, and hypertrophy. Cadmium is a calcium mimetic, and the resulting interference justifies the deleterious effects exerted on many cell signalling pathways, including apoptosis [85]. Lamellar integrity, however, was essentially maintained. This finding aligns with previous observations in gut mucosa [27]. Apoptosis and the activation of DNA repair mechanisms, demonstrated by increased PCNA expression [86], contributed to eliminating damaged cells. Activation by inflammation of the progenitor neural stem cells in the olfactory epithelium’s basal layer cannot be excluded [87].

Connectives also showed damage, in particular, edema and fibrosis of the lamina propria. Collagen deposition is a well-known effect of cadmium exposure [88]. The light microscopy investigations could not discriminate between lamina propria and the basal lamina alterations since the two are in close continuity. Therefore, TEM studies are necessary to verify whether Cd interfered with cell adhesion to the basal membrane or between cells [89]. This interference would have favoured the observed seric infiltration and epithelial detachment from the lamina propria.

Recovery experiments provide further information. The first is that Cd-treated fish performed almost as well as the controls. In the first test, at the first trial, their average time to reach the food was 170 s, much longer than the 110 s employed by controls. In the second test, the difference in the first trial was reduced to 11 s (44 vs. 33 of controls). The prediction was that after recovering, the fish would have taken approximately the same time as the controls in their first trial. However, this was not the case. In our opinion, the exploit performance can be attributed to the fact that in *Danio rerio*, orientation and resulting behaviours also depend on spatial clues. In short, the fish memorised the environment during the first test, even if it did not reach the food. In the second test, place conditioning [90] improved performance six days later, supporting the restored sensory ability. It remains to determine the relevance of visual clues in achieving such goals [91]. In parallel experiments [12], we demonstrated that Cd affects the retina, altering light sensitivity. In this case, recovery was also detected by Muller cells’ regenerative processes.

The restoration of the histological features supports recovery in olfactory lamellae. Positivity for MT and PCNA indicated an initial increase and a subsequent decrease in Cd in the tissues. A lower stress level in the tissues is supported by the decreased number of goblet and rodlet cells and the almost complete disappearance of altered cells. The only unexpected result is the increasing epithelial hypertrophy, a sign of epithelial suffering [92]. A possible explanation is that the metal is still present, at low concentrations, released by tissues such as the liver.

## 5. Conclusions

In conclusion, tests indicate that adults of *Danio rerio* exposed to Cd lose their olfactory ability, and they can fully recover if the insult is short. Much information is still missing, for example, the toxicokinetics of Cd at the concentrations and times tested in the present study. It would also be interesting to determine the concentration of cadmium in different cell types to correlate with damage and, more intriguing, with the apparent incomplete recovery of the epithelium after the period in clean water. Not to be forgotten is the possibility of conducting a parallel study on females and a more in-depth comparison of individual response variability. The collected data, therefore, represent a good basis for future investigations.

We should remember that though increasing attention is paid to the environmental impact of cadmium, contamination by anthropogenic activities continues. The mechanisms underlying olfactory responses in teleost fish must be clarified to protect this essential component of the aquatic environment and the economic advantages humans obtain from correct stock management.

## Data Availability

Data are contained within the article or Supplementary Materials.

## Supplementary Materials

The following supporting information can be downloaded at: https://www.mdpi.com/article/10.3390/biology14010077/s1, Figure S1: Experimental plan adopted for the control and cadmium-treated groups.
