# Supplementary material for: Olfactory Impairment and Recovery in Zebrafish (Danio rerio) Following Cadmium Exposure
Source: Biology (Basel). 2025 Jan 15;14(1):77. doi: 10.3390/biology14010077 (PMC11761868; doi:10.3390/biology14010077)
Supplement: Supplementary file 1 [file biology-14-00077-s001.zip › biology-3392022-supplementary.pdf]

biology

MDPI

Supplementary material

Olfactory Impairment and Recovery in Zebrafish (*Danio rerio*) Following Cadmium Exposure

Experimental design

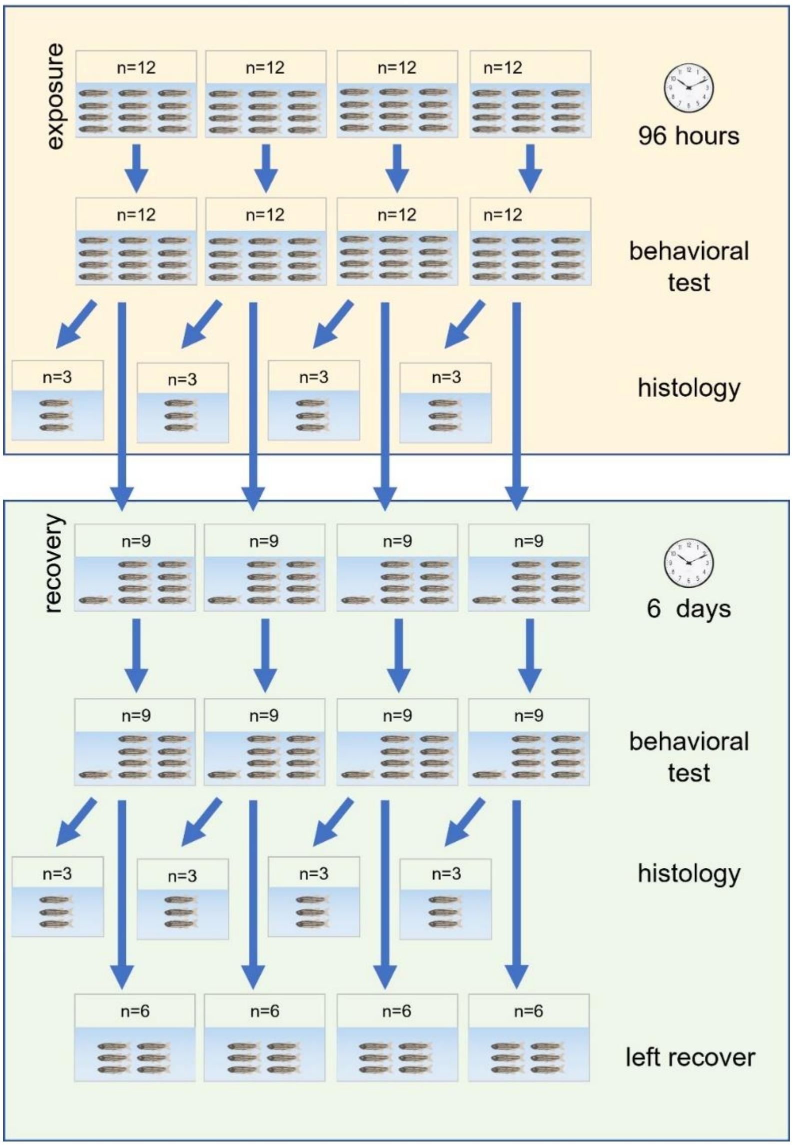

Figure S1. Experimental plan adopted for control and cadmium-treated groups. The fish, controls or to be exposed to cadmium, were allotted into four replicate tanks. After 96 hours, all fish were subjected to the behavioral test, and once completed, three animals per tank were randomly selected and processed for histological analyses. The remaining nine were left to recover in clean water for six days before repeating the behavioural test. In the end, three animals were selected and processed for histological analyses, while the remaining six were left to recover.
